# Supplementary material for: DeepContact: High-throughput quantification of membrane contact sites based on electron microscopy imaging
Source: J Cell Biol. 2022 Aug 5;221(9):e202106190. doi: 10.1083/jcb.202106190 (PMC9361564; doi:10.1083/jcb.202106190)
Supplement: Table S1 — shows performance in ER segmentation using different models. [file JCB_202106190_TableS1.docx]

**Supplementary Table 1. Performance in ER segmentation using different models.**

| Backbone Model | Dice | mIoU |
| --- | --- | --- |
| U-Net | 0.810 | 0.681 |
| FPN | 0.806 | 0.676 |
| LinkNet | 0.802 | 0.670 |
| PSPNet | 0.770 | 0.626 |

Dice, Dice coefficient; mIoU, mean intersection over union
