# Supplementary material for: DeepContact: High-throughput quantification of membrane contact sites based on electron microscopy imaging
Source: J Cell Biol. 2022 Aug 5;221(9):e202106190. doi: 10.1083/jcb.202106190 (PMC9361564; doi:10.1083/jcb.202106190)
Supplement: Table S2 — shows conformities of DeepContact organelle models with manual annotation. [file JCB_202106190_TableS2.docx]

**Supplementary Table 2. Conformities of DeepContact organelle models with manual annotation.**

|  | Mito_match/Mito_manual | ER_match/ER_manual | LD_match/LD_manual |
| --- | --- | --- | --- |
| Mean±SD | 97.63±3.26% | 87.71±5.87% | 98.48±3.71% |

Mito, mitochondria; ER, endoplasmic reticulum; LD, lipid droplet. n = 6, values are presented as mean ± SD. The numbers of segmented organelles are 252-Mito, 210-ER, and 43-LD by manual annotation, and 252-Mito, 210-ER, and 44-LD by DeepContact. The numbers of ER-Mito and LD-Mito contact sites are 220 and 20, respectively.
