# Supplementary material for: DeepContact: High-throughput quantification of membrane contact sites based on electron microscopy imaging
Source: J Cell Biol. 2022 Aug 5;221(9):e202106190. doi: 10.1083/jcb.202106190 (PMC9361564; doi:10.1083/jcb.202106190)
Supplement: Table S3 — shows time consumption comparison between Labelme manual annotation and DeepContact analysis of the ER-Mito MCS of a cultured cell. [file JCB_202106190_TableS3.docx]

**Supplementary Table 3. Time consumption comparison between Labelme manual annotation and DeepContact analysis of the ER-Mito MCS of a cultured cell.**

|  | Preprocessing | Mito  segmentation | ER  Segmentation | Visualization | ER-Mito MCS  quantification | Total |
| --- | --- | --- | --- | --- | --- | --- |
| DeepContact | 0.374 s | 9.647 s | 0.8 s | 5.077 s | 56.196 s | 72.094 s |
| Manual annotation | NA | 16.6±5.5 min. | 53.3±26 min. | NA | NA | NA |

ER, endoplasmic reticulum; Mito, mitochondria; MCS, membrane contact site. NA, not available. n = 10, values are presented as mean or mean ± SD.
