# Supplementary material for: DeepContact: High-throughput quantification of membrane contact sites based on electron microscopy imaging
Source: J Cell Biol. 2022 Aug 5;221(9):e202106190. doi: 10.1083/jcb.202106190 (PMC9361564; doi:10.1083/jcb.202106190)
Supplement: Table S4 — shows time consumption comparison between Labelme manual annotation and DeepContact analysis of the LD-Mito MCS of a cultured cell. [file JCB_202106190_TableS4.docx]

**Supplementary Table 4. Time consumption comparison between Labelme manual annotation and DeepContact analysis of the LD-Mito MCS of a cultured cell.**

|  | Preprocessing | Mito  segmentation | LD  Segmentation | Visualization | LD-Mito MCS quantification | Total |
| --- | --- | --- | --- | --- | --- | --- |
| DeepContact | 0.287 s | 8.586 s | 4.88 s | 4.281 s | 29.582 s | 47.616 s |
| Manual annotation | NA | 16.6±5.5 min. | 4.5±3 min. | NA | NA | NA |

LD, lipid droplet; Mito, mitochondria; MCS, membrane contact site. NA, not available. n = 10, values are presented as mean or mean ± SD.
