# Supplementary material for: DeepContact: High-throughput quantification of membrane contact sites based on electron microscopy imaging
Source: J Cell Biol. 2022 Aug 5;221(9):e202106190. doi: 10.1083/jcb.202106190 (PMC9361564; doi:10.1083/jcb.202106190)
Supplement: Table S5 — shows time consumption comparison between Labelme manual annotation and DeepContact analysis of the ER-Mito MCS of a Sertoli cell in seminiferous epithelial tissue. [file JCB_202106190_TableS5.docx]

**Supplementary Table 5. Time consumption comparison between Labelme manual annotation and DeepContact analysis of the ER-Mito MCS of a Sertoli cell in seminiferous epithelial tissue.**

|  | Preprocessing | Mito  segmentation | ER  Segmentation | PM  segmentation | Visualization | ER-Mito MCS  quantification | Total |
| --- | --- | --- | --- | --- | --- | --- | --- |
| DeepContact | 0.202 s | 1.999 s | 0.828 s | NA | 2.881 s | 38.547 s | 44.458 s |
| Manual annotation | NA | 5.2±4.3 min | 39±18.6 min | 0.3±0.2 min | NA | NA | NA |

ER, endoplasmic reticulum; Mito, mitochondria; PM, plasma membrane; MCS, membrane contact site. NA, not available. n = 10, values are presented as mean or mean ± SD.
